# Giant Bladder Stone: A Case Report

**DOI:** 10.7759/cureus.25439

**Published:** 2022-05-29

**Authors:** Stamatios Katsimperis, Konstantinos Pikramenos, Konstantinos Livadas, Nikolaos Chatzikrachtis, Themistoklis T Bellos

**Affiliations:** 1 Second Department of Urology, National and Kapodistrian University of Athens, Sismanogleio General Hospital, Athens, GRC

**Keywords:** vesical calculi, urinary lithiasis, cystolithotripsy, stone surgery, urology, bladder calculus, cystolithotomy, lithiasis, giant bladder stone

## Abstract

Giant bladder stones, weighing more than 100 g, are a rare entity in western practice, usually associated with bladder outlet obstruction, urinary tract infections, or the presence of intravesical foreign bodies. We present a case of a 53-year-old man with a giant bladder stone weighing 600 g. He underwent suprapubic cystolithotomy, had no major surgical complications, and was discharged with a significantly improved urine flow stream.

## Introduction

Prevalence of urolithiasis in developed countries is between 4% and 20% [1]. Bladder stones appear less common and they usually weigh less than 100 g [1]. Improved healthcare facilities and easy access to them are a reason for this. Thus, the incidence is higher in developing countries [2]. Having a wide presentation ranging from asymptomatic to lower abdominal pain, dysuria, gross hematuria or urinary retention can make the diagnosis challenging [3]. The treatment options are also many. Endourology, open surgery, or even medical therapy can be used. Here, we report a case of a giant bladder stone in an otherwise healthy man who presented to the emergency department with intermittent gross hematuria and dysuria.

## Case presentation

**Figure 1:**
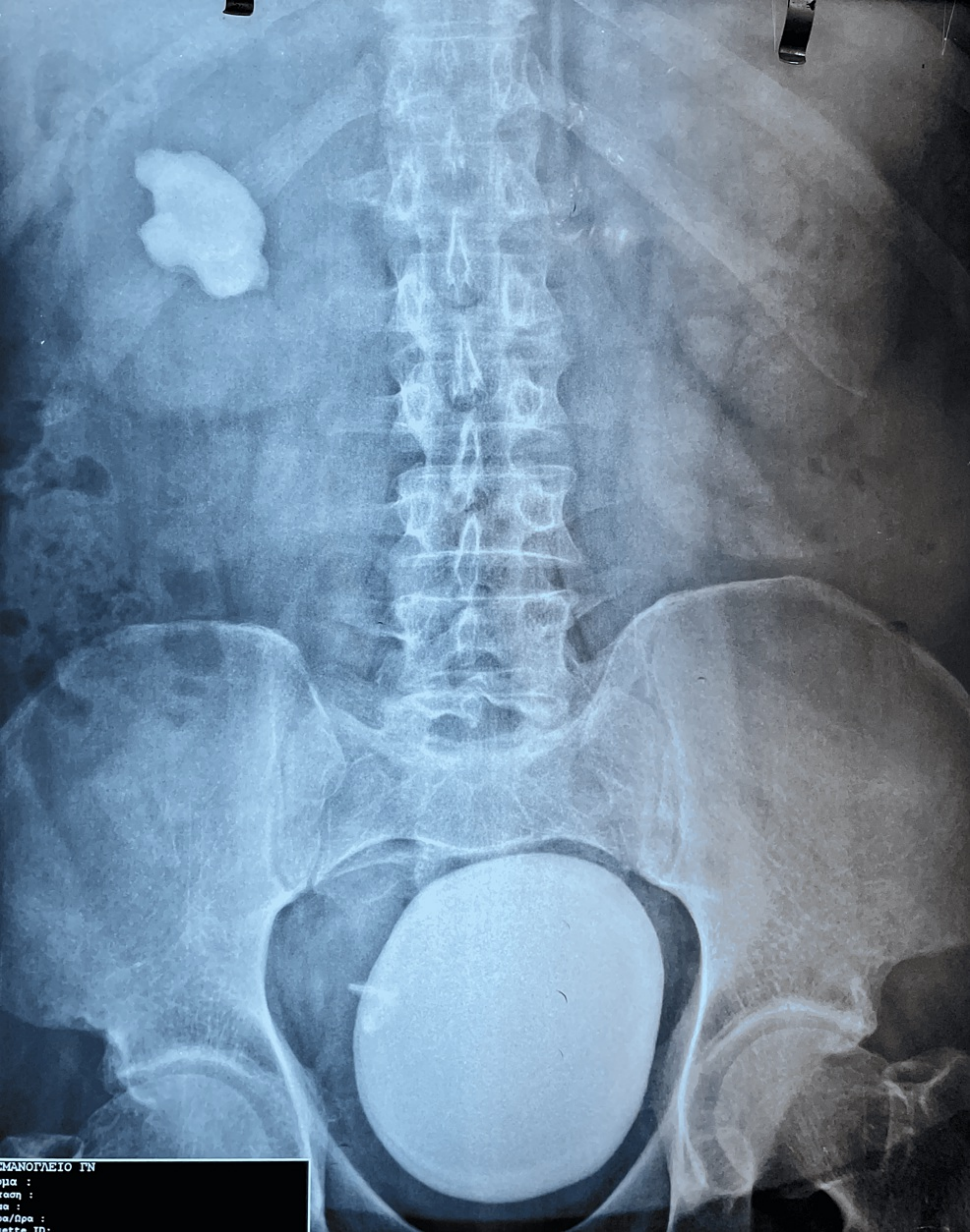
KUB x-ray demonstrating the stone KUB: kidney, ureter, and bladder

A 53-year-old man presented to the emergency department complaining of dysuria and intermittent gross hematuria. From his medical history, he had an open ureterolithotomy surgery on his right ureter 15 years ago. He reported having a pigtail end left in his right ureter from this surgery due to an unsuccessful attempt to remove it and negligence of his own to find a solution. An x-ray showed a bladder stone measuring 9 x 8.7 cm and a stone in his right kidney measuring 4.7 x 3.7 cm (Figure 1). The pigtail remnant he reported was also visible. Mild bilateral hydronephrosis was revealed in the ultrasonography. Urine lab work showed 30-35 red blood cells per high power field and more than 100 pus cells per high power field. His blood examinations were normal. Digital rectal examination revealed a normal prostate.

**Figure 2:**
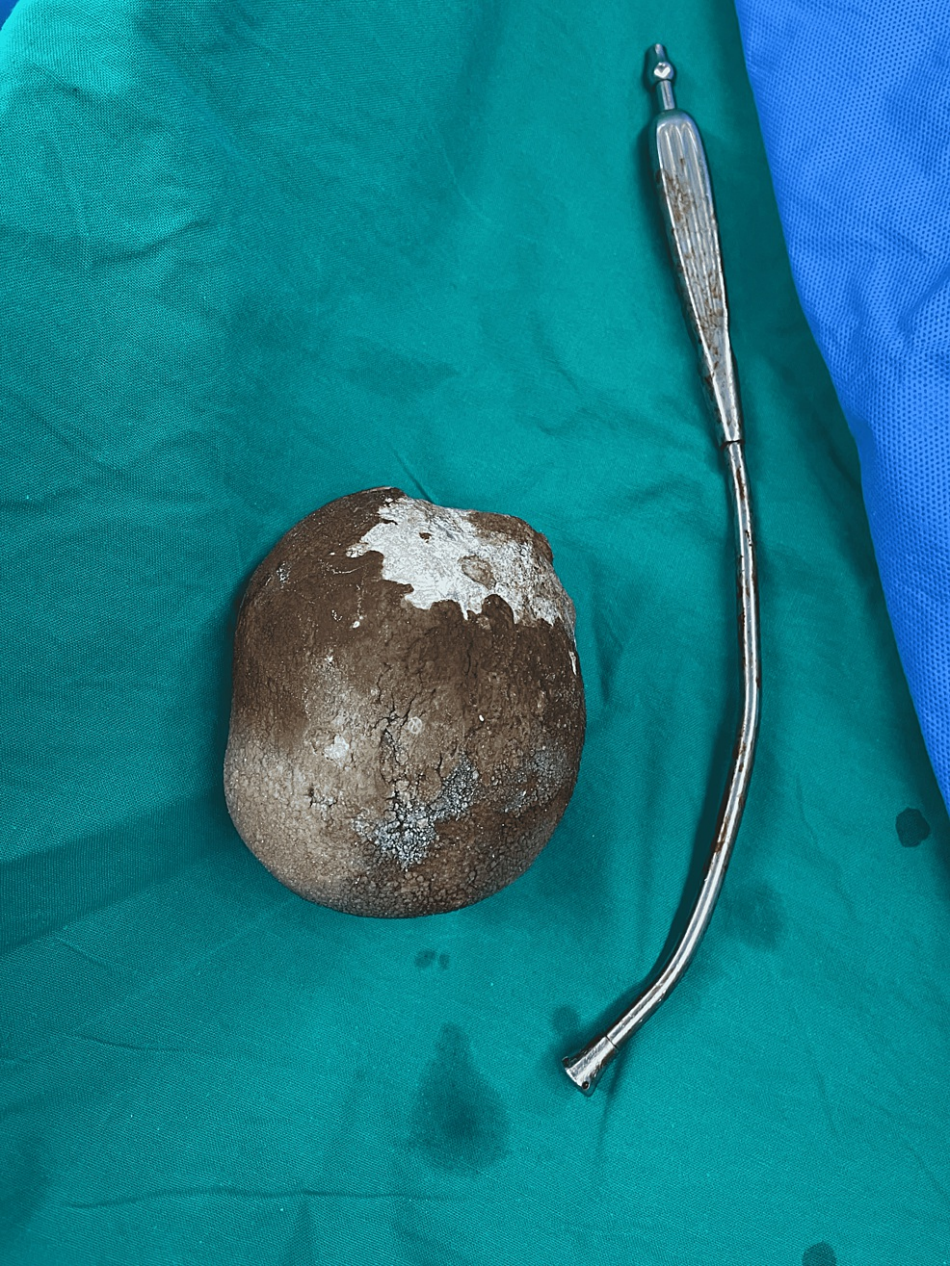
The 600 g bladder stone after its removal

The patient received intravenous ciprofloxacin and underwent open cystolithotomy, which revealed a giant stone weighting 600 g (Figure 2). Both suprapubic catheter and urethral foley catheter were placed. They were removed on postoperative days four and six, respectively, and the patient was discharged on postoperative day eight. On follow-up, he had improved urine flow stream and no hematuria. Two months later, we removed the pigtail mentioned before. We did not take it out during the cystolithotomy because a ureteroscopy was needed to remove it. 

## Discussion

Bladder stones comprise approximately 5% of all cases of urolithiasis [4]. As for those weighing more than 100 g, they are a very rare entity with fewer than 100 cases reported [5]. Most common underlying pathology is voiding dysfunction, chronic infection or presence of intravesical foreign bodies. Benign prostatic enlargement in men [6] and urethral obstruction from pelvic prolapse or cystocele in women are to be blamed [4,7]. Foreign bodies such as forgotten double-J stents or slings that accidentally passed through the bladder are often encrusted and they serve as nidi for stones. Generally, any disorder favoring urinary stasis facilitates stone formation [8]. Usually, the diagnosis is set by ordinary assessment routines such as a plain kidney, ureter, and bladder (KUB) x-ray or an ultrasound scan of the bladder with high sensitivity and specificity rates ranging between 20-83% and 98-100% respectively [9]. When possible, prior to treatment some more exams should be undertaken such as uroflowmetry, urinalysis, urine culture and metabolic assessment, including serum creatinine, calcium, uric acid, sodium and potassium. They will help identify the underlying cause in order to reduce recurrence rates.

Conservative management includes medical treatment with oral urine alkalization for uric acid stones with urine pH > 6.5. Extracorporeal shock wave lithotripsy is another option for the treatment of bladder stones. It is a safe and effective method for patients with high anesthetic risk, patients fearing anesthesia or endoscopic procedures, and patients who have difficulty in positioning. The treatment of choice for bladder stones is usually transurethral cystolithotripsy as it has high stone free rate, shorter hospital stay than other procedures and less pain [10]. However, in selected cases such as very large stones, transurethral cystolithotripsy is not feasible and open cystolithotomy is considered as first line treatment. In contrast to transurethral cystolithotripsy that can be performed under spinal anesthesia, open cystolithotomy requires general anesthesia to be done. This might be a limitation for some patients with certain comorbidities. Other limitations include previous operations or radiation in the abdomen-pelvis that might make the surgery impracticable. Despite these limitations, open cystolithotomy, when feasible, is very effective and lacks complications such as urethral strictures that one may face after transurethral cystolithotripsy [11]. Patient also becomes total stone-free in most cases in one session, something that could not be achieved with transurethral cystolithotripsy in such big stones. Because of the strong correlation of bladder stones with benign prostatic hyperplasia, laparoscopic cystolithotomy performed in combination with simple prostatectomy using either traditional laparoscopy or with robotic assistance has also been described as another option [12,13]. In our case, due to the massive size of the stone, laparoscopy would not offer any benefit compared to the open approach as a big incision was needed for the extraction of the stone.

## Conclusions

Giant bladder stones are not common today. They can present with symptoms that do not predispose them to such a rare entity. Treatment modality for each patient differs with respect to the size of the stone and feasibility of the surgery. Usually, open cystolithotomy is the most effective treatment. In the current era of endourology and minimal invasive surgery, open cystolithotomy still has a place in the urological armamentarium.
